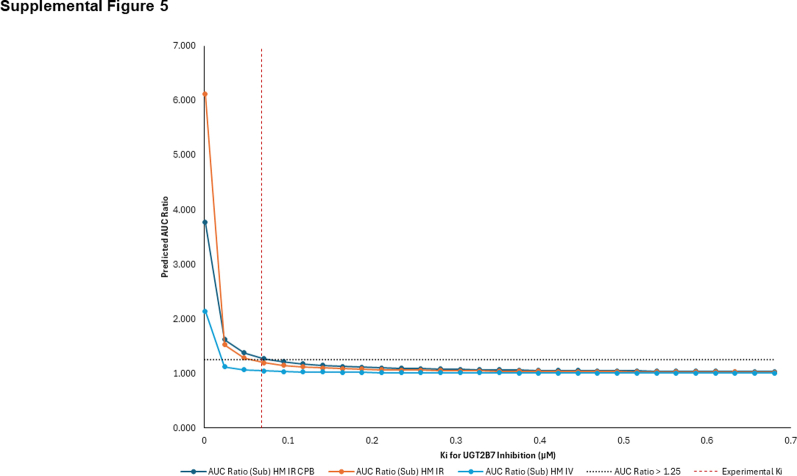# UGT2B7-mediated drug–drug interaction between cannabinoids and hydromorphone

**DOI:** 10.1016/j.dmd.2025.100135

**Published:** 2025-07-24

**Authors:** Shelby Coates, Keti Bardhi, Mengqi Zhao, Philip Lazarus

**Affiliations:** 1 Department of Pharmaceutical Sciences, College of Pharmacy and Pharmaceutical Sciences, Washington State University, Spokane, Washington; 2 Division of Molecular Biosciences, Department of Pharmaceutical Sciences, School of Pharmacy and Pharmaceutical Sciences, University at Buffalo, Buffalo, New York

**Keywords:** Cannabidiol, Drug–drug interactions, Hydromorphone, Physiologically based pharmacokinetic modeling, Tetrahydrocannabinol, UDP-glucuronosyltransferase

## Abstract

Hydromorphone is a highly potent opioid used to treat severe chronic pain. It is metabolized primarily by UDP-glucuronosyltransferase (UGT)2B7 to form the inactive hydromorphone-3-glucuronide. Given that previous studies have shown that the major cannabinoids, *Δ*^9^-tetrahydrocannabinol (THC) and cannabidiol (CBD), inhibit several UGT enzymes, the objective of the present study was to determine the inhibitory potential of major cannabinoids and their metabolites on UGT-mediated hydromorphone metabolism. To evaluate the potential for cannabis-induced drug interactions, cannabinoids and their metabolites were screened as potential inhibitors against hydromorphone glucuronidation in pooled human liver microsomes and microsomes from cells overexpressing recombinant UGT2B7. IC_50_ values were determined for cannabinoids that inhibited hydromorphone glucuronidation by 50% and *K*_i_ values for those that exhibited an IC_50_ < 100 *μ*M in human liver microsomes. Potent inhibition of hydromorphone metabolism was observed for THC, 11-hydroxy (OH)-THC, CBD, and 7-OH-CBD, with *K*_i_ values ranging from 0.068 to 1.01 *μ*M after correction for nonspecific cannabinoid binding. Differences in inhibition were observed for the UGT2B7^268Tyr^ variant compared with the wildtype UGTB7^268His^ isoform for several cannabinoids. Static modeling indicated that THC, 11-OH-THC, CBD, and 7-OH-CBD would result in drug interactions in vivo after inhalation and oral consumption of THC and CBD (>1.25-fold increase in hydromorphone exposure), with physiologically based pharmacokinetic predictive models indicating that CBD would cause a 20%–30% increase in hydromorphone exposure in healthy and cirrhotic individuals. These data suggest that major cannabinoids such as CBD will cause moderate drug–drug interactions with hydromorphone in humans.

**Significance Statement:**

This study indicates that major cannabinoids and their metabolites found in the plasma of cannabis users inhibit UGT2B7-mediated hydromorphone metabolism in vitro. It further demonstrates the potential for in vivo inhibition of hydromorphone metabolism by cannabinoids and their metabolites, indicating the potential for drug–drug interactions upon concomitant use of hydromorphone and cannabis or hydromorphone together with individual cannabinoids like *Δ*^9^-tetrahydrocannabinol and cannabidiol.

## 1 Introduction

Hydromorphone is an opioid analgesic that is commonly used to treat moderate to severe chronic pain.^1^ Hydromorphone is typically prescribed after a patient becomes tolerant to another opioid (eg, morphine) and is generally reserved as a second-line treatment due to its high potency (5–10 times more potent than morphine)^2^, ^3^, ^4^ and addiction risk.^5^^,^^6^ Hydromorphone is primarily metabolized by UDP-glucuronosyltransferase (UGT)2B7 to form its major inactive metabolite, hydromorphone-3-glucuronide (HM3G).^7^, ^8^, ^9^, ^10^ Although HM3G is inactive, it is thought to cause some of the negative side effects associated with hydromorphone use.^9^^,^^11^^,^^12^

Because opioids have a high risk for addiction, patients have turned to alternative sources for their pain management. Retrospective studies have found that 40%–60% of those surveyed use cannabis to treat their unspecified chronic pain.^13^^,^^14^ Furthermore, 50% of adults who are prescribed opioids to manage their chronic pain also use cannabis.^15^^,^^16^ Cannabis contains numerous phytochemicals (cannabinoids), including the 2 commonly known cannabinoids—*Δ*^9^-tetrahydrocannabinol (THC), which elicits the high associated with cannabis use,^17^, ^18^, ^19^ and cannabidiol (CBD), which also have a wide range of pharmacologic effects including pain relief, anti-inflammatory effects, and antiepileptic effects.^17^^,^^20^, ^21^, ^22^, ^23^, ^24^ The major cannabinoids (THC and CBD) undergo metabolism after consumption to multiple metabolites. THC is metabolized to an active metabolite, 11-hydroxy (OH)-*Δ*^9^-THC,^25^ which is metabolized to 11-nor-9-carboxy (COOH)-*Δ*^9^-THC,^25^^,^^26^ which is then subsequently glucuronidated to form 11-nor-*Δ*^9^-THC-carboxylic acid glucuronide.^27^ CBD is metabolized to an active metabolite, 7-OH-CBD,^28^^,^^29^ which is subsequently metabolized to 7-COOH-CBD.^29^^,^^30^ The systemic exposure of both major cannabinoids and their metabolites is high, with their metabolites often exceeding parent exposure by ≥1.25-fold.^31^ Owing to the increasing use of cannabis to treat a myriad of medical conditions including chronic pain, the potential inhibitory effect cannabinoids and their metabolites may have on drug-metabolizing enzymes (DMEs) should be investigated to ensure these compounds are safe when used in combination with other agents.

Previous in vitro studies have shown that major cannabinoids and their metabolites inhibit both phase I and phase II DMEs,^32^, ^33^, ^34^, ^35^, ^36^ including UGTs 1A6, 1A9, 2B4, and 2B7, and have shown that several cannabinoids and/or their metabolites are likely to cause a drug–drug interaction (DDI) in vivo based upon static mechanistic modeling.^35^, ^36^, ^37^, ^38^ The goal of the present study was to explore whether cannabinoids induce a UGT-mediated DDI with hydromorphone as a substrate; to use in vitro to in vivo extrapolation (IVIVE) models, including physiologically based pharmacokinetic (PBPK) models; to predict the clinical significance of a potential cannabinoid mediated DDI; and to determine whether genetic variation in DMEs important in hydromorphone metabolism affects this interaction.

## 2 Materials and methods

### 2.1 Chemicals and materials

THC, 11-OH-THC, 11-COOH-THC, CBD, 7-OH-CBD, and 7-COOH-CBD were obtained from Cayman Chemicals or Sigma-Aldrich after obtaining approval from the Drug Enforcement Administration. Hydromorphone and HM3G standards were purchased from Sigma-Aldrich; HM3G-D_3_ internal standard was unavailable for purchase, and morphine-3-glucuronide-D_3_ (also purchased from Sigma-Aldrich) was used as a surrogate. Pooled human liver microsomes (HLM; 50 subjects, mixed biological sex) were purchased from Sekisui Xenotech. Ultralow-binding microcentrifuge tubes as well as high-performance liquid chromatography–grade ammonium formate, methanol, and formic acid were purchased from Thermo Fisher Scientific. Ketoconazole was purchased from Sigma-Aldrich, and UDP glucuronic acid (UDPGA) was purchased from Cayman Chemical. ChromatoPur bovine serum albumin (BSA) was purchased from MB Biomedicals. Dulbecco’s Modified Eagles Medium, Dulbecco’s phosphate-buffered saline, and geneticin (G418) were purchased from Gibco, and premium grade fetal bovine serum was purchased from Seradigm. The ACQUITY UHPLC HSS T3 (1.8 *μ*M × 2.1 mm × 100 mm) column used for ultrahigh-performance liquid chromatography-mass spectrometry (UHPLC-MS) was purchased from Waters.

### 2.2 Generation of UGT2B7 overexpressing cell lines

The UGT2B7^268His^ and UGT2B7^268Tyr^ overexpressing human embryonic kidney (HEK)293 cell lines have been previously developed and described^39^ and UGT2B7 sequences were verified by Sanger sequencing. The parent HEK293 cell line used in this study was purchased from American Type Culture Collection in 2015 and authenticated by American Type Culture Collection in 2019 using short-tandem repeat polymorphism analysis. *Mycoplasma* species was not detected in these cells in 2021.

For relative UGT quantification, equal amounts of microsomal protein (20 *μ*g) were loaded on 10% SDS-PAGE and UGT protein quantity was determined by western blot analysis using an anti–UGT2B-HRP antibody in a 1:2500 dilution. Calnexin was used as a loading control for microsomal fractions, with an anti–calnexin-HRP antibody used in a 1:1000 dilution for all analyses. ImageJ software (http://rsb.info.nih.gov/ij; NIH) was used to perform densitometry analysis, and the relative expression of each UGT-containing microsomal preparation was used for normalization in glucuronidation activity assays.

### 2.3 Enzyme kinetic constants determinations in HLM and microsomes from recombinant UGT2B7^268His^ and UGT2B7^268Tyr^ overexpressing cells

Glucuronidation reactions (final volume = 50 *μ*L) contained 25–60 *μ*g of total microsomal protein (recombinant [r]UGT2B7^268His^, rUGT2B7^268Tyr^, and HLM), 25 mmol/L Tris buffer, 2 mmol/L MgCl_2_, 2% BSA, and varying concentrations of hydromorphone (0.01–5000 *μ*M). Reactions were initiated by the addition of 4 mM UDPGA and incubated for 1 hour at 37 °C. Each experiment had a negative control (UDPGA negative) reaction. Kinetic constants were determined from 3 independent experiments.

### 2.4 Inhibition screenings of cannabinoids and their metabolites as inhibitors of UGT2B7

Microsomal membrane fractions were prepared from HEK293 cells overexpressing the rUGT2B7^268His^ and rUGT2B7^268Tyr^ variants by differential centrifugation as previously described.^39^^,^^40^ Total microsomal protein concentrations were determined using the BCA assay per the manufacturer’s instructions. UGT2B7 inhibition assays (final volume = 50 *μ*L) were performed in reactions contained 20–100 *μ*g of total microsomal protein (rUGT2B7^268His^, rUGT2B7^268Tyr^, and HLM), 25 mmol/L Tris buffer, 2 mmol/L MgCl_2_, 2% BSA, and 400 *μ*M hydromorphone (ie, the known *K*_M_ for UGT2B7 against hydromorphone^7^). An individual cannabinoid or cannabinoid metabolite was added as a potential inhibitor at 2 different concentrations, 10 and 100 *μ*M. Microsomes were preincubated with alamethicin (50 *μ*g/mg) on ice for 15 minutes before incubation. Reactions were initiated by the addition of 4 mM UDPGA and incubated for 1–1.5 hours at 37 °C. Each experiment had a positive control reaction containing 10 or 100 *μ*M of probe inhibitor (ketoconazole), which was added instead of cannabinoid. The relative activity of a given reaction was measured against a reaction containing only vehicle (3% methanol) and no inhibitor.

To reduce the nonspecific binding of cannabinoids to labware, low-bind microcentrifuge tubes were used for all reactions. Additionally, 2% BSA was added to sequester inhibitory long-chain unsaturated fatty acids that are known to inhibit the activity of UGT2B7^41^^,^^42^ and increase cannabinoid solubility.^25^^,^^43^ Reactions were terminated with the addition of 50 *μ*L of ice-cold methanol containing internal standards (morphine-3-glucuronide-D_3_). Samples were then centrifuged for 30 minutes at 17,000*g* at 4 °C. Supernatants were then collected and run on the UHPLC-MS/MS as described further.

Inhibition assay conditions were optimized for overexpressing cell lines and HLM for both reaction time and microsomal protein added. Optimal conditions were based on the following criteria: (1) metabolite formation was linear with enzyme concentration and time, (2) substrate depletion was <20% during the incubation, and (3) metabolite formation detection with the UHPLC-MS/MS method was reproducible.

### 2.5 IC_50_ determinations

For the cannabinoids and metabolites that exhibited ≥50% inhibition of HM3G formation at either the 10 or 100 *μ*M cannabinoid concentrations, IC_50_ values were determined in UGT2B7^268His^ and UGT2B7^268Tyr^ overexpressing HEK293 cell microsomes and in HLM. The assay parameters were the same as those described earlier for the inhibition screenings, using a range of 10–12 cannabinoid inhibitor concentrations (0.1–250 *μ*M). IC_50_ values were determined from 3 independent experiments. An IC_50_ cutoff of <100 *μ*M was used to determine whether inhibition constant (*K*_i_) experiments would be performed in HLM.

### 2.6 Inhibition type and K_i_ determinations

Data observed from the IC_50_ studies were used to determine the substrate and inhibitor concentration ranges for *K*_i_ experiments in HLM. The hydromorphone experimental concentration range consisted of 3 concentrations between 120 *μ*M and 1.2 mM (0.3- to 3-fold the *K*_m_ of UGT2B7 for hydromorphone glucuronidation), whereas the cannabinoid inhibitor concentration range consisted of 5 concentrations (0-fold and 0.3- to 3-fold the experimentally determined IC_50_ for a given cannabinoid in HLM. *K*_i_ data was used to determine the type of reversible inhibition exerted on UGT2B7 by each cannabinoid inhibitor.

### 2.7 Correction for cannabinoid nonspecific binding

Cannabinoids and their metabolites are highly lipophilic and have extensive nonspecific binding to labware and microsomal protein.^44^ To account for the nonspecific binding of cannabinoids to both labware and protein, fraction unbound determinations previously reported by our laboratory^32^^,^^33^ were used in the present studies; given their structural similarity, the 11-OH-THC *f*_u,inc_ value was used as a surrogate for 11-COOH-THC, 7-OH-CBD, and 7-COOH-CBD. IC_50_ values were corrected for nonspecific binding by cannabinoids $\left({\text{IC}}_{50,u}\right)$ by calculating the unbound fraction in the incubation for individual cannabinoids (*f*_u,inc_) in either HLM or HEK293 microsomes as follows:

(1) $${\text{IC}}_{50,u}={\text{IC}}_{50}\times f_{u,\text{inc}}$$

*K*_i_ values were corrected for nonspecific binding by cannabinoids in a similar fashion as the IC_50_ values as follows:

(2) $$K_{i,u}=K_{i}\times f_{u,\text{inc}}$$

### 2.8 UHPLC-MS/MS analysis

UHPLC-MS/MS was performed with mobile phases consisting of 5 mM ammonium formate and 0.1% UHPLC-MS/MS grade formic acid (buffer A), and methanol containing 0.1% UHPLC-MS/MS grade formic acid (buffer B). A UHPLC-HSS T3 (1.8 *μ*M × 2.1 × 100 mm) column with a flow rate of 0.40 mL/min was used to separate HM3G and hydromorphone as follows: 30 seconds at 100% A, 30 seconds at 95% A, 4 minutes at 25% A, 30 seconds at 5% A, and reequilibration for 1.5 minutes at 100% A. The injection volume was 1–5 *μ*L with a column temperature of 30 °C. MS/MS detection was performed in a Waters ACQUITY XEVO TQD instrument in MRM ESI+ mode. The MS/MS scans were performed using the following mass transitions: HM3G (m/z 462.2000 > 286.2000), hydromorphone (m/z 286.2000 > 185.1000), and morphine-3-glucuronide-D_3_ (m/z 465.2000 > 289.2000), respectively. The collision energy was optimized to 32 V for both HM3G and morphine-3-glucuronide. A cone voltage of 30 V and 0.044 second dwell time resulted in high sensitivity detection of hydromorphone, HM3G, and morphine-3-glucuronide-D_3_. The desolvation temperature was 500 °C, with 1000 L/h of nitrogen gas. Glucuronide metabolite retention times observed in the enzymatic incubations were compared with the deuterated glucuronide metabolite internal standard retention times. Because a deuterated internal standard of HM3G was not available at the time of this study, morphine-3-glucuronide-D_3_ was used as a surrogate internal standard because they have the same molecular weight. Metabolite concentrations were quantified using Targetlynx software (version 4.1; Waters Acquity) by interpolation from matrix-matched standard curve (0.0036–15 ppm) prepared using standard and deuterated internal standard. The curve fitting, weighting, and accuracy of the standard curve was a linear fit (weighting = 1/*x* and *R*^2^ = 0.996, respectively). Assay precision was validated by repeated (3–5 times) sample quantification, with a coefficient of variation of <10%. No matrix effect was observed in the inhibition reactions when compared with internal or reference standard alone. In-source dissociation of glucuronides was not observed. Possible carryover from previous MS runs was tested routinely (after every 3–5 reactions); none was observed. Reaction sample stability was greater than 1 week (7 days) when stored at 4 °C.

### 2.9 Data analysis

Kinetic parameters were determined from the Michaelis-Menten equation using GraphPad Prism 7.04 software (GraphPad Software). Relative maximum reaction rates (*V*_max_) were calculated as picomoles per minute per milligram of protein, with values normalized to wildtype UGT2B7^268His^ microsomal protein as determined by western blot analysis using ImageJ software as described earlier. All reported values represent the results (eg, mean + SD) of 3 independent experiments. The activity of the UGT2B7^268Tyr^ variant isoform was compared with its corresponding wildtype isoform using the Student’s *t*-test. A two-tailed *P* value of <.05 was considered the threshold for statistical significance.

Data were exported and analyzed using Excel (Microsoft). The amount of metabolite formation at each concentration of cannabinoid relative to the no inhibitor control (ie, percent metabolite formation) was calculated using the ratio of peak area of sample/peak area of internal standard to obtain the peak area of metabolite with and without inhibitor ratios. Ratios were subsequently normalized to percent of control (no inhibitor), which were calculated using the peak area of metabolite with inhibitor ratio/peak area of metabolite without inhibitor ratio. Metabolite peak area was normalized to respective internal standard peak area.

IC_50_ values were calculated by plotting percent metabolite formation (metabolite peak area with inhibitor normalized to respective internal standard peak area divided by normalized metabolite peak area without inhibitor) versus the log concentration of each cannabinoid inhibitor evaluated using GraphPad Prism 7.04 software. IC_50_ curves were constrained to 100 and 0 for top and bottom of the curves, respectively.

*K*_i_ values determined in HLM were calculated by plotting the rate of reaction (pmol/min per milligram of protein) against substrate concentration (*μ*M) using GraphPad Prism 7.04 software. The rate of reaction and substrate concentration were replotted using Lineweaver-Burk plots to linearize the data. The *y*-intercept and slope of each replicate (*n* = 3) were replotted to determine the type of inhibition (competitive, uncompetitive, noncompetitive, and mixed). A *P* value of <.05 was used as a cutoff to indicate that either the slope of the replotted slope or *y*-intercepts was statistically significantly different from zero indicating 1 of the 4 inhibition types.

### 2.10 Static mechanistic IVIVE

Static mechanistic modeling was used to initially determine the potential for a DDI to occur in vivo after coadministration of hydromorphone and either THC or CBD. *K*_i_ values generated in HLM experiments were used to perform static mechanistic modeling as recommended by the Food and Drug Administration (FDA).^31^

The DDI potential from inhibition of DMEs was assessed using static mechanistic modeling as recommended by the FDA,^31^ which assesses the potential for a DDI in vivo by comparing the area under the concentration time curve (AUC) in the presence and absence of inhibitor to generate the AUC ratio (AUCR) as follows:

(3) $$\text{AUCR}=\left(\frac{1}{A_{g}\times \left(1-F_{g}\right)+F_{g}}\right)\times \left(\frac{1}{A_{h}\times f_{m}+\left(1-f_{m}\right)}\right)$$

where *F*_g_ is the fraction available after intestinal metabolism and was set to 1 as recommended by the FDA^31^ and *f*_*m*_ is the specific enzyme contribution to metabolism of hydromorphone to its respective metabolites and set to 0.95 (total hydromorphone glucuronidation).^45^ As recommended by the FDA, an AUCR cutoff of ≥1.25 was used to indicate the potential for a DDI in vivo.^31^

Equation 4 defines *A*_g_ as the effect of reversible inhibitions in the intestine on substrate drug (morphine). [*I*]_g_ is the inhibitor concentration in the intestine and is defined in eq. 5, where *F*_a_ is the fraction absorbed after oral administration, which was set to 1 as recommended by the FDA^31^ for inhalation and 1 for THC and CBD after oral administration, respectively. *K*_a_ is the first-order absorption rate constant in vivo and was set to 0.02.^46^

(4) $$A_{g}=\frac{1}{1+\frac{{\left[I\right]}_{g}}{K_{i,u}}}$$

(5) $${\left[I\right]}_{g}=\frac{F_{a}\times K_{a}\times \text{Dose}}{Q_{\text{en}}}$$

Equation 6 defines *A*_h_ as the effect of reversible inhibitions in the liver on the substrate drug (hydromorphone):

(6) $$A_{h}=\frac{1}{1+\frac{{\left[I\right]}_{h}}{K_{i,u}}}$$

[*I*]_h_ is the inhibitor concentration in the liver and is defined as follows:

(7) $${\left[I\right]}_{h}=f_{u,p}\times \left(C_{\max}+\frac{F_{a\;}\times F_{g}\times K_{a}\times \text{Dose}}{Q_{h\;}\times R_{b}}\right)$$

where *f*_u,p_ is the unbound fraction of inhibitor in plasma, and *C*_max_ is the maximal total (free and bound) inhibitor concentration in the plasma.

For the static mechanistic modeling, hepatic blood flow (*Q*_h_) and enterocyte blood flow (*Q*_en_) were set to 1500 and 300 mL/min, respectively, and the blood-to-plasma concentration ratio (*R*_B_) was set to 0.4.^47^ A range of THC and CBD doses (THC: 20–160 mg; mean, 70 mg; CBD: 19–2000 mg; mean, 700 mg)^36^ were used to simulate average low and high doses of THC or CBD through both oral and inhalation routes of administration (Supplemental Table 1). Based upon static mechanistic modeling, the cannabinoid with the highest potential for a DDI in vivo with hydromorphone was investigated further through PBPK modeling.

### 2.11 PBPK model development and validation

PBPK models were developed using Simcyp software version 23.1. Basic physiochemical and pharmacokinetic (PK) parameters used to develop the models are listed in Table 1 (hydromorphone) and Supplemental Table 2 (CBD). The predicted steady-state volume of distribution was used to develop a whole-body hydromorphone PBPK model.^48^^,^^49^ Although hydromorphone is purported to be a substrate of P-glycoprotein, this mechanism was not included in the PBPK model as the hydromorphone transport by P-glycoprotein is not clearly established.^50^^,^^51^

**Table 1:** Key physiochemical and system-specific input parameters for the development of physiologically based pharmacokinetic model for hydromorphone using Simcyp v23

Parameters	Value
Physiochemical properties^a^	
Molecular weight (g/mol)	285.34
Log *P*	1.63
p*K*_*a*_ = 1	10.11
p*K*_*a*_ = 2	8.59
Blood binding^b^	
Blood-to-plasma ratio	1.04
Fraction unbound in plasma	0.92
Absorption	
First-order absorption model	
*f*_*a*_^c^	0.67
*k*_*a*_^c^ (1/h)	1.04
*f*_ugut_^d^	1
*Q*_gut_^c^ (L/h)	26.12
Distribution: full PBPK model	
*V*_ss_^e^ (L/kg)	2.81
Elimination	
Clearance type: enzyme kinetic (HLM)^f^ HM3G pathway/enzyme UGT2B7 CL_int_ (*μ*L/min per milligram of protein)	84.7
Additional clearance	
CL_R_ (L/h)^g^	8.232

ADAM, advanced dissolution, absorption and metabolism; CL_R_, renal clearance; *f*_*a*_, fraction absorbed from dosage form; *f*_ugut_, fraction unbound in the gut; HLM, human liver microsomes; *k*_*a*_, first-order absorption rate constant; p*K*_*a*_, acid dissociation constant; log *P*, log of the partition coefficient of a solute between octanol and water; *Q*_gut_, gut blood flow; *V*_ss_, volume of distribution at steady state.

a Physiochemical data were obtained from the ChEMBL database (https://www.ebi.ac.uk/).

b Dilaudid package insert.

c Simcyp predicted using parameter estimation.

d Assumed to be 1.

e Simcyp prediction using method 3 (Rogers and Rowland method + ion membrane permeability).

f Calculated by the middle-out approach from the Dilaudid package insert.

g CL_R_ was calculated based on *f*_*e*_, fraction excreted unchanged in urine (0.07; Dilaudid package insert).

Hydromorphone’s clearance profile was developed using a middle-out approach using both in vitro and in vivo data, where UGT2B7-mediated glucuronidation was assumed to be the sole metabolic pathway responsible for hydromorphone hepatic clearance.^7^, ^8^, ^9^, ^10^ The in vivo intrinsic hepatic clearance (CL_int,H_) was backcalculated using in vivo plasma clearance (CL_IV_) using the dispersion model and was further optimized to recapitulate the observed in vivo clearance (117.6 L/h).^52^ The CL_int,H_ was assigned to UGT2B7 because it being responsible for >95% of hydromorphone metabolism.^52^

The first-order absorption model was used to predict the oral hydromorphone PK profile. Both the fraction absorbed (*F*_a_) and absorption rate constant (*K*_a_) for hydromorphone was predicted using Simcyp parameter estimation to recapitulate the observed hydromorphone PK profiles of the training data sets. Brain tissue concentrations were predicted using the permeability-limited brain model with the passive permeability-surface area product (PSB) on the blood–brain barrier and the PSB on the brain–cerebrospinal fluid (CSF) barrier (PSC) determined as follows:

(9) $$\text{PSB}=P_{\text{app},A\to B}\times \text{SA}$$

where SA is the human brain microvasculature surface area (15–25 m^2^) and $P_{\text{app},A\to B}$ is the apparent permeability of hydromorphone optimized to recapitulate the observed CSF hydromorphone concentrations. PSC is expected to be one-half of the PSB (half of the surface area).^53^

The hydromorphone model was developed and validated using the healthy adult population within Simcyp. A minimum of 400 participants were simulated to mimic the population variability within the observed data. The age, sex, number of subjects, dose, route of administration, dosing regimen, and prandial state in the simulations were matched to the corresponding in vivo study. The model was also validated in the hepatically impaired population for oral formulations using existing Simcyp model (Child-Pugh B) for moderate hepatic impairment. Model predictive performance was evaluated by comparing the predicted AUC and *C*_max_ to the observed in vivo data that were not used as training data sets. The model was considered to be validated if the ratio of predicted AUC and *C*_max_ to observed AUC and *C*_max_ were within 0.5- to 2-fold range. The model was further analyzed visually by comparing whether the observed data points fell within the 90% confidence intervals (CIs) of the simulated concentration–time curve (WebPlotDigitizer v4.7; https://automeris.io/WebPlotDigitizer). Model precision was also analyzed by determining the mean relative deviation (MRD) and geometric mean fold error (GMFE) of both AUC and *C*_max_ predicted-to-observed ratios using the following equations:

(10) $$\text{MRD}=10^{\sqrt{\frac{\sum_{i=1}^{k}{\left(\log_{10}\hat{c_{i}}-\log_{10}c_{i}\right)}^{2}}{k}}}$$

where $c_{i}$ is the *i*th observed plasma concentration, $\hat{c_{i}}$ predicted plasma concentration corresponding to the *i*th observed plasma concentration, and *k* the number of observed values; and

(11) $$\text{GMFE}=10^{\sqrt{\frac{\sum_{i=1}^{m}\left|\log_{10}\left(\frac{\hat{p_{i}}}{p_{i}}\right)\right|}{m}}}$$

where $p_{i}$ is the observed AUC_last_ or *C*_max_ value of study *i*, $\hat{p_{i}}$ the corresponding predicted AUC_last_ or *C*_max_ value of study *i*, and *m* the number of studies. A previously developed and validated PBPK model for CBD^54^ was used to investigate the risk of the potential DDI between hydromorphone and CBD.

### 2.12 DDI model validation

Sensitivity analysis was performed to evaluate predictive model performance by decreasing the *K*_i_ values of CBD against morphine glucuronidation by 1- to 10-fold. It is expected that with decreasing *K*_i_ values, the AUCR would increase.

### 2.13 DDI prediction

DDI trials were simulated with 400 subjects (20 subjects × 20 trials) in healthy adults, with an equal proportion of males and females and age range of 20–40 years. CBD oral doses (1500 mg every 12 hours) were administered for 7 days, while hydromorphone administration was set as a single intravenous and oral dose, which were coadministered with CBD on the morning of day 7. Intravenous and immediate-release tablet hydromorphone were both set at 8 mg. The magnitude of the DDI is presented as the hydromorphone AUC ratio with versus without CBD (AUC_inhibtor_/AUC_no inhibitor_).

## 3 Results

### 3.1 Enzyme kinetic determinations

**Supplementary Figure 1:**
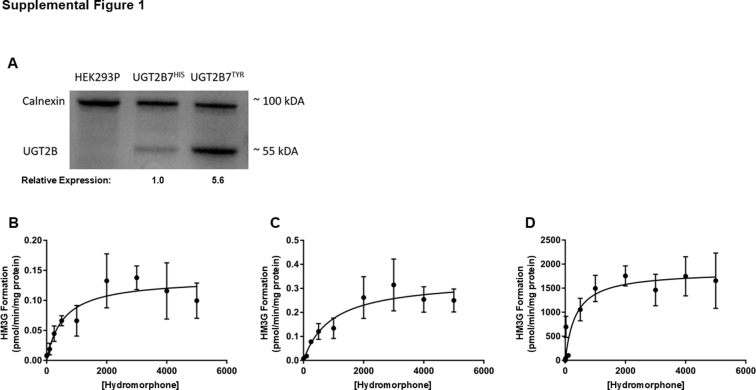


UGT2B7 is a polymorphic enzyme with the most common variant containing a single nucleotide change at amino acid 268 from histidine to tyrosine.^55^^,^^56^ In the present study, HEK293 cell lines overexpressing either the rUGT2B7^268His^ or rUGT2B7^268Tyr^ isoforms were used. UGT2B7 enzyme expression in the 2 cell lines was compared using western blot analysis to normalize for UGT2B7 protein expression in each HEK293 cell line (Supplemental Fig. 1). To determine whether glucuronidation activity differences were observed between the 2 UGT2B7 isoforms against hydromorphone, kinetic studies were performed. The *K*_m_ and CL_int_ for hydromorphone glucuronidation varied between microsomes containing the 2 UGT2B7 variants, with a significantly (*P* = .014) lower *K*_m_ and a significantly (*P* = .011) higher CL_int_ for rUGT2B7^268His^ microsomes (545 ± 116 *μ*M and 0.25 ± 0.06 nL/min per milligram of UGT2B protein, respectively) than those observed for rUGT2B7^268Tyr^ microsomes (961 ± 79 *μ*M and 0.06 ± 0.02 nL/min per milligram of UGT2B protein, respectively) (Table 2). The *K*_m_ in HLM (from a pool of 50 people) approached that observed in rUGT2B7^268His^ microsomes (361 ± 109 *μ*M).

**Table 2:** Kinetic analysis of UGT2B7 variants against hydromorphone

	*K*_m_ (*μ*M)^a^	*V*_max_ (pmol/min per milligram of protein)^a^	CL_int_ (nL/min per milligram of UGT2B protein)^a^^,^^b^	Relative Activity^c^
UGT2B7^268His^	545 ± 116	0.14 ± 0.03	0.254 ± 0.06	1.0
UGT2B7^268Tyr^	961 ± 79	0.061 ± 0.01	0.065 ± 0.02	0.25
HLM	361 ± 109	1864 ± 237	5.44 ± 0.91^c^	

a *K*_m_, *V*_max_, and CL_int_ are expressed as the mean ± SD of 3 independent experiments.

b CL_int_ = *μ*L/min per milligram of liver microsomal protein, calculated using the *K*_m_ and *V*_max_ values from the individual replicates.

c The relative activity is equivalent to the CL_int_ ratio of UGT2B7^268Tyr^ to UGT2B7^268His^ microsomes.

### 3.2 Inhibition screening and inhibition mechanism determination

**Fig. 1:**
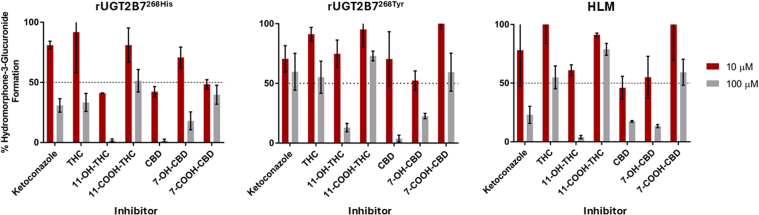
Inhibition screening assays against HM3G formation in microsomes from HEK293 cell lines overexpressing rUGT2B7^268His^ or rUGT2B7^268Tyr^ or in HLM. All activities were compared with control reactions without inhibitor. Ketoconozole was added as a positive control inhibitor for UGT2B7. Red bars, 10 *μ*M ketoconozole or cannabinoid; gray bars, 100 *μ*M ketoconozole or cannabinoid. Data shown are the mean ± SD of 3 experiments.

Inhibition screenings of THC, 11-OH-THC, 11-COOH-THC, CBD, 7-OH-CBD, and 7-COOH-CBD as potential inhibitors of hydromorphone metabolism in rUGT2B7^268His^ microsomes showed that 100 *μ*M THC, 11-COOH-THC, and 7-COOH-CBD inhibited HM3G formation by 67%, 49%, and 60%, respectively (Fig. 1A). Slightly lower levels of inhibition were observed with 100 *μ*M of THC, 11-COOH-THC, and 7-COOH-CBD (45% 27%, and 41% inhibition of HM3G formation, respectively) in rUGT2B7^268Tyr^ microsomes (Fig. 1B). Greater inhibition was observed with 100 *μ*M 11-OH-THC, CBD, and 7-OH-CBD, with respective decreases in HM3G formation of 98%, 98%, and 82% observed in rUGT2B7^268His^ microsomes (Fig. 1A) and 87%, 96%, and 77% in rUGT2B7^268Tyr^ microsomes (Fig. 1B). Inhibition screening results were confirmed in pooled HLM, with 100 *μ*M 11-OH-THC, CBD, and 7-OH-CBD inhibiting HM3G formation by 96%, 83%, and 87%, respectively, while 100 *μ*M THC, 11-COOH-THC, and 7-COOH-CBD exhibited only minimal or moderate inhibition of HM3G formation (Fig. 1C). Similar to that observed for microsomes from both rUGT2B7 isoforms, 11-COOH-THC did not show appreciable inhibition of HM3G formation in HLM.

**Supplementary Figure 2:**
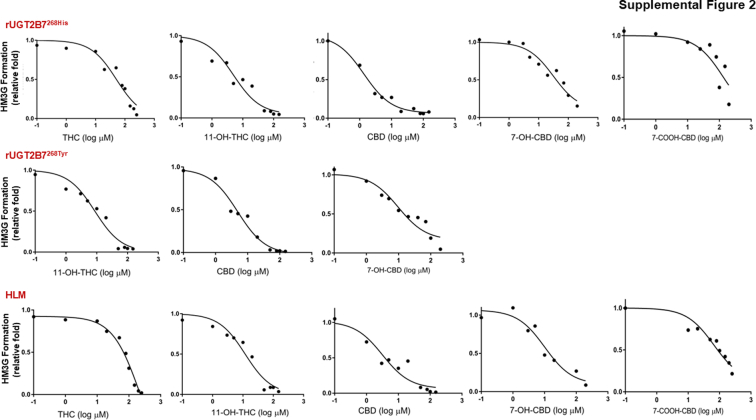


The concentration-dependent inhibition of hydromorphone metabolism by THC, 11-OH-THC, CBD, and 7-OH-CBD was further established in rUGT2B7 microsomes as well as in HLM by determining IC_50_ and corresponding IC_50,u_ values for each cannabinoid that inhibited HM3G formation by at least 50% at 100 *μ*M in the inhibition screening assays. Representative IC_50_ curves are shown for each cannabinoid in recombinant UGT2B7-overexpresing microsomes and HLM in Supplemental Fig. 2. CBD exhibited the most potent inhibition of HM3G metabolite formation in rUGT2B7-containing microsomes and HLM, with IC_50,u_ values of 0.074 ± 0.010 *μ*M, 0.15 ± 0.02 *μ*M, and 0.14 ± 0.04 *μ*M for rUGT2B7^268His^ microsomes, rUGT2B7^268Tyr^ microsomes, and HLMs, respectively (Table 3).^32^^,^^33^ Further, 11-OH-THC and 7-OH-CBD also exhibited potent inhibition of HM3G formation in UGT2B7-containing microsomes and HLM, with IC_50,u_ values of 0.36 ± 0.03 *μ*M and 1.39 ± 0.77 *μ*M for rUGT2B7^268His^ microsomes, 0.84 ± 0.19 *μ*M and 1.26 ± 0.93 *μ*M for rUGT2B7^268Tyr^ microsomes, and 1.08 ± 0.14 *μ*M and 0.95 ± 0.25 *μ*M for HLMs, respectively. IC_50,u_ values showed weak or moderate inhibition of HM3G formation for THC and 7-COOH-CBD in rUGT2B7 microsomes and HLM.

**Table 3:** IC_50,u_ values (*μ*M) for the inhibition of UGT2B7-mediated hydromorphone metabolism to hydromorphone-3-glucuronide by individual cannabinoids

Enzyme source	THC	11-OH-THC	CBD	7-OH-CBD	7-COOH-CBD
	*μM*^a^	*μM*^a^	*μM*^a^	*μM*^a^^,^^b^	*μM*^a^^,^^b^
rUGT2B7^268His^	2.92 ± 0.26	0.36 ± 0.03	0.074 ± 0.010	1.39 ± 0.77	5.54 ± 3.67
rUGT2B7^268Tyr^	ND	0.84 ± 0.19	0.15 ± 0.02	1.26 ± 0.93	ND
HLM	2.50 ± 0.58	1.08 ± 0.14	0.14 ± 0.04	0.95 ± 0.25	>10

r, recombinant enzyme from overexpressing cell microsomes; ND, not determined.

a IC_50,u_ values are the mean ± SD. IC_50_ (in *μ*M) corrected for nonspecific binding with previous fraction unbound (*f*_u,inc_) values determined by Nasrin et al.^32^^,^^33^

b The *f*_u,inc_ for 11-OH-THC was used as a surrogate value for calculating IC_50,u_.

**Fig. 2:**
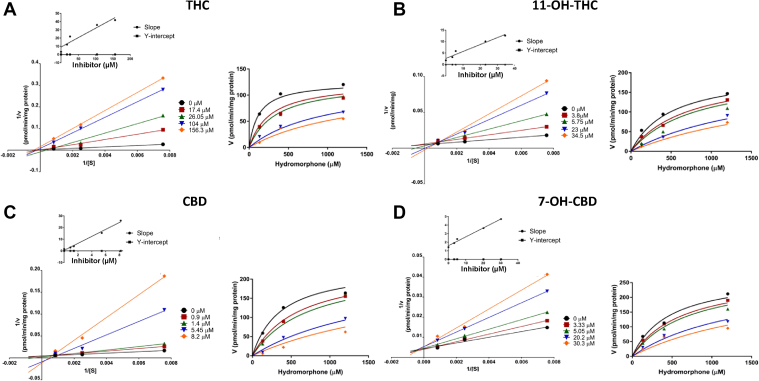
Concentration-dependent inhibition of UGT2B7-mediated hydromorphone glucuronidation by the individual cannabinoids: (A) THC, (B) 11-OH-THC, (C) CBD, and (D) 7-OH-CBD. Each panel consists of the corresponding Lineweaver-Burk plot (left) inlayed with the replot of the *y*-intercepts and slopes from each inhibitor concentration, and Michaelis-Menten curves (right) used to estimate the *K*_i_ based upon either competitive or mixed (11-OH-THC) models. Curves denote single representative plots for each inhibitor substrate concentration.

The inhibitor constant (*K*_i_) and inhibition type for each cannabinoid that exhibited inhibition was determined for UGT2B7-mediated metabolism of hydromorphone in HLM. A replot of the slopes and *y*-intercept generated from the Lineweaver-Burk plot (double reciprocal of velocities vs substrate concentration) indicated that THC, CBD, and 7-OH-CBD inhibited UGT2B7 in a competitive manner (Fig. 2). Interestingly, 11-OH-THC was shown to have mixed type inhibition, although visually the type of inhibition approaches competitive type inhibition (Fig. 2). The THC, CBD, and 7-OH-CBD K_i,u_ (corrected for nonspecific binding of the cannabinoids) values (0.17 ± 0.02 *μ*M, 0.068 ± 0.003 *μ*M, and 0.74 ± 0.12 *μ*M, respectively) were calculated using the competitive model (Table 4),^32^^,^^33^ while the 11-OH-THC *K*_i,u_ value (1.01 ± 0.18 *μ*M) was determined using a mixed inhibition model; the *α* value (*α* >> 1) obtained when using GraphPad further suggested that 11-OH-THC inhibition approached a competitive inhibition model.

**Table 4:** Experimentally determined type of reversible inhibition and *K*_i,u_ (*μ*M) values of individual cannabinoids for the inhibition of UGT2B7-mediated hydromorphone metabolism in HLM

Cannabinoid	Inhibition Type	*K*_i,u_^a^
		*μM*
THC	Competitive	0.17 ± 0.02
11-OH-THC	Mixed	1.01 ± 0.18
CBD	Competitive	0.068 ± 0.003
7-OH-CBD	Competitive	0.74 ± 0.12^b^

a *K*_i,u_ values are the *K*_i_ values corrected for nonspecific binding with previous fraction unbound (*f*_u,inc_) values as determined in Nasrin et al.^32^^,^^33^

b The *f*_u,inc_ for 11-OH-THC was used as a surrogate for calculating *K*_i,u_.

### 3.3 Mechanistic static modeling to predict drug interactions

**Fig. 3:**
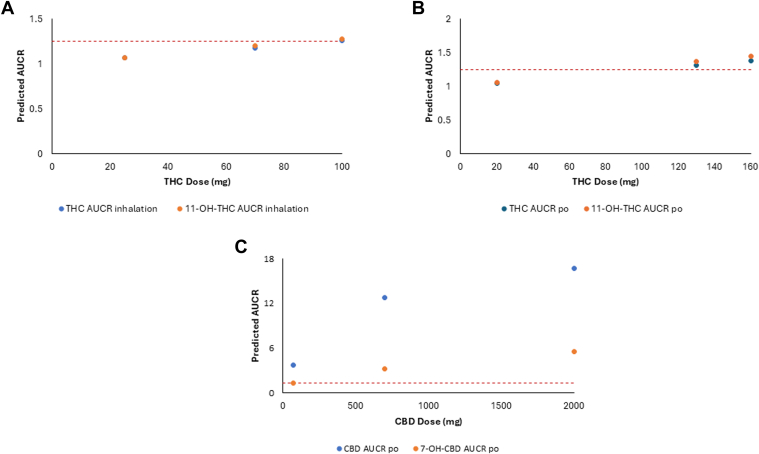
Drug interaction risks predicted via mechanistic static models associated with THC or CBD use from low to high doses of major cannabinoids and their metabolites: (A) THC and 11-OH-THC after inhalation; (B) THC and 11-OH-THC after oral administration; and (C) CBD and 7-OH-CBD after oral administration. Red dashed line shows the FDA area under the curve ratio (AUCR) cutoff, indicating a drug interaction of 1.25.

Mechanistic static modeling populated with the experimentally determined *K*_i,u_ values (Table 4) were used to predict in vivo DDI for THC, 11-OH-THC, CBD, and 7-OH-CBD when THC or CBD was coadministered with hydromorphone. Models were calculated by comparing hydromorphone exposure in the presence or absence of cannabinoid, with several significant (AUCR > 1.25) interactions observed. After inhalation of 100 mg of THC, the predicted AUCR for THC and 11-OH-THC after coadministration with hydromorphone was 1.26 and 1.28, respectively, while after oral administration of 130 mg of THC, the predicted AUCR for THC and 11-OH-THC after coadministration with hydromorphone was 1.31 and 1.37, respectively (Table 5).^36^ After inhalation of 19 mg of CBD, the predicted AUCR for CBD was 2.15 after coadministration of hydromorphone, while after oral administration of 70 mg of CBD, the predicted AUCR for CBD and 7-OH-CBD when coadministered with hydromorphone was 3.7 and 1.26, respectively (Table 5). For larger oral doses of CBD, the predicted AUCR for CBD and 7-OH-CBD when coadministered with hydromorphone was 12.8 and 3.19, respectively, for 700 mg CBD and 16.7 and 5.54, respectively, for 2000 mg CBD (Table 5). Static mechanistic modeling performed with increasing doses of both THC and CBD indicated increased AUCR proportionally with dose, suggesting that with increasing dosing of either THC or CBD, the risk for a DDI also increased (Fig. 3).

**Table 5:** Prediction of clinical UGT-mediated cannabis hydromorphone drug interactions via mechanistic static modeling after inhaled or oral doses of THC and CBD

Cannabinoid	Dose^a^mg	Route of Administration	Hydromorphone AUCR
THC	20	Oral	1.05
	130	Oral	1.31^b^
	160	Oral	1.38^b^
	25	Inhalation	1.07
	70	Inhalation	1.18
	100	Inhalation	1.26^b^
11-OH-THC	20	Oral	1.06
	130	Oral	1.37^b^
	160	Oral	1.45^b^
	25	Inhalation	1.07
	70	Inhalation	1.20
	100	Inhalation	1.28^b^
CBD	70	Oral	3.70^b^
	700	Oral	12.8^b^
	2000	Oral	16.7^b^
	19	Inhalation	2.15^b^
7-OH-CBD	70	Oral	1.26^b^
	700	Oral	3.19^b^
	2000	Oral	5.54^b^
	19	Inhalation	ND

ND, not determined.

a Doses and *C*_max_ used to predict AUCR were reported from Bansal et al.^36^ The doses used for modeling 11-OH-THC and 11-COOH-THC were from administered doses of THC, while 7-OH-CBD was modeled using administered doses of CBD.

b Values indicate AUCR values that are ≥1.25 as recommended by the FDA.

### 3.4 PBPK model for hydromorphone after intravenous and oral administration

**Supplementary Figure 3:**
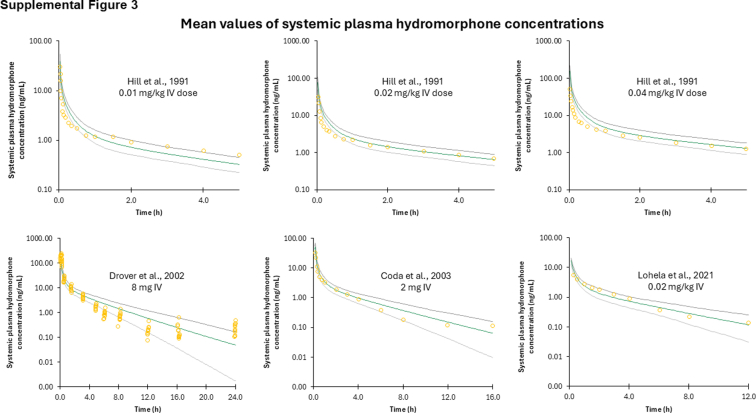


The PBPK model–predicted hydromorphone plasma concentration–time profile following intravenous administration was comparable with the observed in vivo data used as training and validation datasets (Supplemental Fig. 3). Most of the in vivo data points fell within the 90% CIs (5th–95th CI) of the simulated data (Supplemental Table 3). The predicted-to-observed AUC GMFE and MRD ratios were 1.99 and 1.82, respectively. These results indicate successful development of a PBPK model for hydromorphone after intravenous administration that can simulate clinically observed PKs of hydromorphone.

**Supplementary Figure 4:**
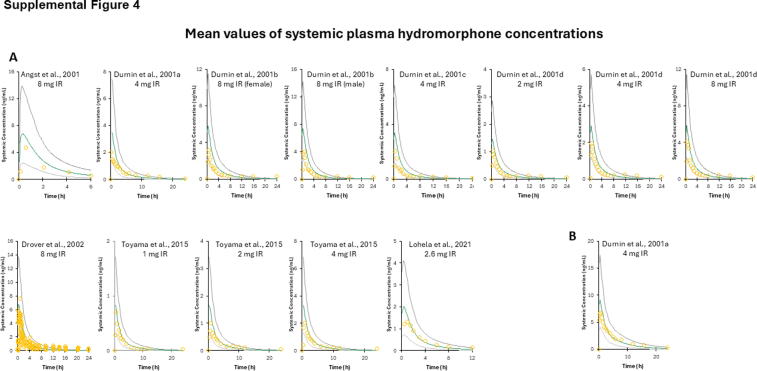


The PBPK model–predicted hydromorphone plasma concentration–time profile following oral administration with immediate-release formulation was comparable with the observed in vivo data used as training and validation datasets (Supplemental Fig. 4). Most of the in vivo data points fall within the 90% CIs (5th–95th CI) of the simulated data (Supplemental Table 3). The predicted-to-observed AUC and *C*_max_ GMFE and MRD ratios were 1.89 and 2.29, and 1.98 and 2.41, respectively. While the MRD average ratio for both the AUC and *C*_max_ exceeded 2, the majority of the individual MRD values for each model are <2 and the other acceptance criteria (AUC and *C*_max_ ratio and visual fitting) are met. Similar to that described earlier for intravenous administration of hydromorphone, these data indicate successful development of a PBPK model for hydromorphone after oral administration that can simulate clinically observed PKs of hydromorphone.

The PBPK model–predicted hydromorphone plasma concentration–time profile following immediate-release oral administration in moderate hepatically impaired adults was comparable with observed data (Supplemental Fig. 4). Most of the observed data points fall within the 90% CIs (5th–95th CI) of the simulated data. The predicted-to-observed AUC and C_max_ GMFE and MRD ratios were 1.88 and 1.19, and 1.16 and 1.01, respectively (Supplemental Table 3).

### 3.5 PBPK model for DDI simulation with coadministration of hydromorphone and CBD

The PBPK model for substrate (hydromorphone) and the previously validated PBPK model for inhibitor (CBD)^55^ were combined to predict the magnitude of the potential DDI arising from inhibition of hydromorphone glucuronidation by CBD in a virtual healthy population and in a virtual population of adults with moderate cirrhosis. Owing to the lack of clinical data investigating this potential DDI for model validation, sensitivity analysis was used to evaluate model robustness; this was performed by decreasing the *K*_i_ value (1- to 10-fold) to simulate an increase in the ratio of simulated hydromorphone AUC. As shown in Supplemental Fig. 5, reduced *K*_i_ values for CBD against hydromorphone glucuronidation resulted in an expected increase in the simulated hydromorphone AUC in both intravenous and oral administration models.

**Fig. 4:**
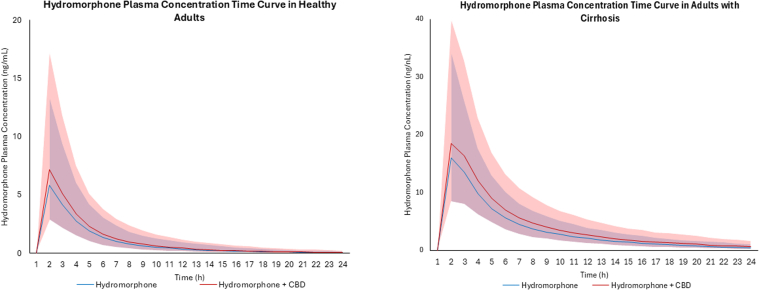
PBPK-predicted hydromorphone plasma concentration–time curves in healthy and cirrhotic populations after immediate-release oral administration of 8 mg hydromorphone with coadministration of CBD (1500 mg twice daily for 7 days) with 90% CIs. The blue line represents hydromorphone alone and the red line hydromorphone with CBD, with 90% CIs indicated by blue and red shading, respectively.

**Fig. 5:**
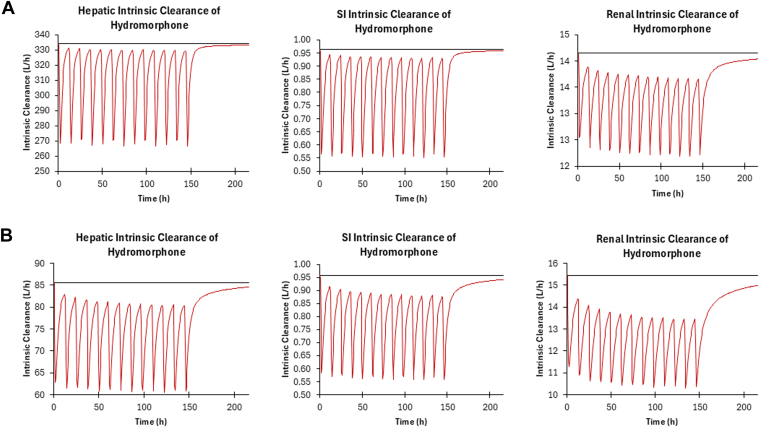
Hydromorphone intrinsic clearance (CL_int_) after CBD administration (1500 mg twice daily for 7 days) with 8 mg of immediate-release hydromorphone in (A) healthy and (B) cirrhotic adults. There was a 20.2%, 42.7%, and 13.9% decrease in hepatic, small intestine, and renal hydromorphone CL_int_ in healthy adults and a 29.3%, 41.8%, and 33.3% decrease in hepatic, small intestine, and renal hydromorphone CL_int_, respectively, with CBD vs hydromorphone alone. The black line represents hydromorphone alone and the red line hydromorphone with CBD. SI, small intestine.

Based on virtual trials with healthy subjects coadministered CBD (1500 mg twice daily for 7 days) and hydromorphone (8 mg) administered intravenously, an increase in hydromorphone exposure in healthy populations was not predicted, with a predicted AUCR for this combination of 1.06 (Table 6). Based on virtual trials with healthy subjects coadministered CBD (1500 mg twice daily for 7 days) and immediate-release oral hydromorphone (8 mg), a moderate increase in hydromorphone exposure (22%) was observed (Fig. 4), with a predicted AUCR of 1.22 (Table 6). Based on virtual trials with subjects with moderate hepatic impairment, coadministration of CBD (1500 mg twice daily for 7 days) and 8 mg immediate-release oral hydromorphone led to a similar moderate increase in hydromorphone exposure (25%) (Fig. 4), with a predicted AUCR of 1.25 (Table 6). Based on (intrinsic organ clearance) CL_int_ in the liver, kidney, and the gut, the DDI is predicted to have the largest impact in the small intestine in both healthy and cirrhotic populations (Fig. 5).

**Table 6:** Simulated hydromorphone pharmacokinetic parameters following intravenous or oral administration with and without CBD in virtual populations of healthy and cirrhotic subjects^a^

	Dose *mg*	AUC *ng/h per mL*	90% CI	*C*_max_*ng/mL*	90% CI
**Sim-Healthy Hydromorphone Intravenous**					

Hydromorphone	8	93.79	92.43–95.18	-	-
Hydromorphone+CBD^b^		99.42	97.93–100.93	-	-
Ratio^c^		1.06		-	
Hydromorphone IR					
Hydromorphone	8	15.69	15.08–16.33	5.91	5.65–6.19
Hydromorphone+CBD^b^		19.15	18.36–19.97	7.33	7.00–7.68
Ratio^c^		1.22		1.24	

**Sim-Cirrhosis**					

Hydromorphone IR					
Hydromorphone	8	62.67	60.75–64.65	16.31	15.71–16.92
Hydromorphone+CBD^b^		78.44	75.86–81.11	19.08	18.37–19.81
Ratio^c^		1.25		1.17	

AUC, areas under the plasma concentration–time curves; *C*_max_, maximum plasma concentration; IR, immediate release.

a Data presented as the geometric mean and 90% CI.

b CBD dosing was 1500 mg twice daily for 7 days with hydromorphone administered on the morning of Day 6.

c AUC ratio or *C*_max_ ratio as determined by AUC_inhibitor_/AUC_no inhibitor_ or *C*_max,inhibitor_/*C*_max_,_no inhibitor_, respectively.

**Fig. 6:**
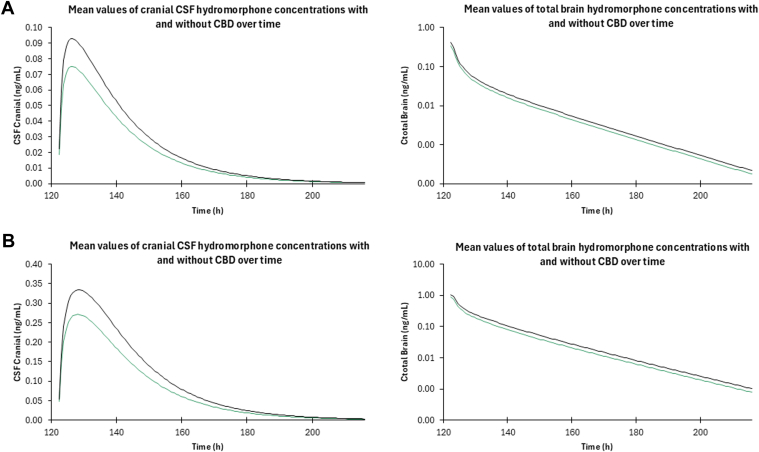
PBPK-predicted hydromorphone brain and CSF concentration–time curves in (A) healthy and (B) cirrhotic populations after immediate-release oral administration of 8 mg hydromorphone with coadministration of CBD (1500 mg twice daily for 7 days). The green line represents hydromorphone alone and the black line hydromorphone with CBD.

Concentrations of hydromorphone within the brain were also analyzed during concomitant administration of hydromorphone and CBD to examine the potential pharmacodynamic effect of the potential DDI. Because hydromorphone has a narrow therapeutic index, changes in brain concentrations can lead to serious adverse events such as increased respiratory depression and overdose.^57^ Predicted hydromorphone brain tissue concentrations after concomitant administration of CBD and hydromorphone in healthy and cirrhotic populations increased by 24% and 27%, respectively (Fig. 6).

## 4 Discussion

To our knowledge, this is the first comprehensive investigation of the inhibitory effects of major cannabinoids and their metabolites against UGT2B7-mediated glucuronidation and metabolism of hydromorphone. Results from this study show that major cannabinoids and their active metabolites exhibit strong inhibition of UGT2B7 metabolism of hydromorphone in vitro and that PBPK modeling suggests that CBD inhibits hydromorphone’s UGT2B7-mediated metabolism to a clinically significant level (AUCR ≥ 1.25-fold). The PBPK model–predicted hydromorphone *C*_max_ and AUC after both single intravenous and oral administration in both healthy and cirrhotic populations fell within predefined acceptance criteria (Supplemental Figs. 3 and 4; Supplemental Table 3). These model validation results indicate successful development of a PBPK model to predict hydromorphone PK without inhibitor and can be applied in drug interaction models like those used in this study. Further, 11-OH-THC and 7-OH-CBD exhibited more moderate inhibition of hydromorphone UGT-mediated metabolism when compared with CBD. THC and the cannabinoid metabolites 11-COOH-THC and 7-COOH-CBD did not exhibit or exhibited weak to moderate inhibitory activity against hydromorphone metabolism. These studies are consistent with previous studies demonstrating an inhibitory effect of several major cannabinoids and their metabolites on UGT2B7-mediated metabolism using different substrates^33^^,^^37^^,^^38^ and with the fact that the *K*_i,u_ values determined for cannabinoids against hydromorphone metabolism in the present study were similar to those determined against morphine metabolism.^37^

Interestingly, the static mechanistic modeling used in the present study better predicted the in vivo potential DDI risk than our previous work with morphine,^37^ with static modeling with morphine over-predicting the DDI risk compared with the subsequent risk predicted by PBPK models.^37^ Although the DDI risk predicted for hydromorphone in the present study was still over-predicted by static mechanistic modeling compared with PBPK model predictions, the hydromorphone static modeling was more accurate than morphine static mechanistic models, an effect illustrated by differences in calculated *K*_i_ values: the *K*_i_ for CBD against UGT2B7 in the presence of hydromorphone (0.068 *μ*M) was 3-fold lower than the *K*_i_ for CBD against UGT2B7 in the presence of morphine.^37^ This suggests that, in the case of hydromorphone and CBD, the concentrations of CBD are high enough to meet and sustain the *K*_i_ concentration to exert a longer-lasting inhibitory effect on UGT2B7-mediated hydromorphone metabolism than that on morphine metabolism.

Although clinical studies have shown that the UGT2B7∗2 allele did not have a significant effect on hydromorphone PKs and disposition,^58^^,^^59^ a UGT2B7-specific genetic effect may influence the inhibition of hydromorphone glucuronidation by cannabinoids. The inhibitory effect of 11-OH-THC and CBD against UGT2B7∗2 were 2- to 3-fold lower than that observed with UGT2B7∗1, with the IC_50,u_ values for UGT2B7∗1 by cannabinoids approaching or within physiological levels achieved after consuming cannabis, THC, or CBD.^60^^,^^61^ Furthermore, THC and 7-COOH-CBD had little to no inhibitory effect on hydromorphone metabolism for the UGT2B7∗2 variant compared with that on UGT2B7∗1 where a moderate or weak effect was observed. These data suggest that individuals homozygous for the UGT2B7∗2 allele may be less likely to exhibit significant inhibition of hydromorphone metabolism compared with individuals homozygous for the UGT2B7∗1 allele.

Durnin et al^62^ found that dosage reduction is needed in patients with liver cirrhosis who are prescribed hydromorphone to avoid toxicity and adverse side effects. This dosage reduction may also become increasingly important if the patient is also concomitantly using CBD or cannabis. This drug–disease interaction is not only affected by the increase in hydromorphone levels owing to the DDI induced by CBD but also affected by the known hepatoxic effects of chronic CBD usage.^63^^,^^64^ Durnin et al^62^ observed that in cirrhotic patients, there was a 4-fold increase in plasma concentrations and overall exposure of hydromorphone compared with that in healthy individuals.^62^ A 4-fold increase in exposure to an opioid, especially one as potent as hydromorphone, is likely to increase the risk for adverse side effects to occur, necessitating dosage adjustments in this population. Furthermore, the results obtained in the present study suggest that there are compensatory mechanisms for hydromorphone elimination in cirrhotic patients as liver function is decreased in this population. This was in fact observed in the predictive DDI models in moderate hepatically impaired subjects, where the renal contribution increased and the subsequent renal CL_int_ decreased in the presence of CBD compared with those in healthy populations.

The DDI models predict a clinically relevant 20%–30% increase in hydromorphone exposure in both healthy and cirrhotic populations taking CBD. Hydromorphone is a potent opioid^1^, ^2^, ^3^, ^4^ that has a narrow therapeutic window that, when exceeded, causes serious adverse side effects like respiratory depression and fatal overdoses.^1^ The 20%–30% increase is clinically significant not only in regard to the FDA cutoff for clinically relevant drug interactions (AUCR ≥ 1.25-fold)^31^ but also in context of the narrow therapeutic window for hydromorphone safety and efficacy. The DDI simulations in the present study suggested that predicted hydromorphone brain concentrations also increase 20%–30% in the presence of CBD in both healthy and hepatically impaired subjects. Such an increase in hydromorphone brain exposure would likely necessitate a decrease in dose and/or frequency of administration to ensure patient safety, suggesting that clinical monitoring of patients taking both hydromorphone and CBD is needed to ensure hydromorphone efficacy and safety for the patient.

The current study has a few limitations. Only 1 UGT2B7 allelic variant was studied and did not include the UGT2B7∗3 allele, which is prevalent in the Japanese population.^65^ In addition, the brain PBPK model described in this study is a prediction based on data without any available clinical data in the literature to compare with. The hydromorphone CSF concentrations are from studies where lumbar epidurals were given to patients, and this route of administration was not able to be modeled using Simcyp.^66^ In addition, hydromorphone PSB and PSC values were predicted based upon the predicted $P_{\text{app},A\to B}$ from Simcyp.

Another potential limitation of the present study was that while CBD’s active metabolite, 7-OH-CBD, also showed potent inhibition of hydromorphone UGT2B7-mediated metabolism, it was not incorporated into the inhibitor model. The DDI potential shown with our current PBPK models is therefore likely an underprediction of the in vivo risk owing to not incorporating the inhibitory effects of this and potentially other longer-lasting cannabinoid metabolites. Furthermore, the long-term administration of CBD was not considered within the PBPK models owing to a lack of clinical studies investigating the PKs of long-term CBD use with typical chronic CBD dosing. Moreover, because of the lack of literature on the effect of UGT2B7 genotype on oral immediate-release hydromorphone PKs, we were unable to incorporate genotype differences in inhibitory effect that we observed in vitro into the PBPK models. The current PBPK model also did not incorporate the potential pharmacodynamic effects with concomitant use of CBD with hydromorphone. Future models can be developed to investigate the potential pharmacodynamic changes associated with CBD use with hydromorphone and the risk for DDI. Finally, PBPK DDI models are only predictive because there are no available clinical studies investigating this DDI; clinical studies may be necessary to more fully elucidate the severity of the DDI between hydromorphone and CBD.

In lieu of the recent International Council for Harmonization Drug Interaction Studies M12 guidance (ICHM12), investigating potential drug interactions involving UGTs is warranted when glucuronidation is a primary route of elimination.^67^ There is no current guidance for cutoffs to determine DDI risk using basic models like there is for CYP enzymes. It is recommended that the same cutoff is applied to UGTs as is applied to CYPs, which is what was performed with the static mechanistic modeling described in the present study.^67^ However, a 20%–30% increase in object drug exposure is not a significant DDI as compared to CYP DDIs—the therapeutic index of the drug must be considered when looking at severity of DDI. For narrow therapeutic indexed drugs like opioids (ie, hydromorphone), a 30% increase in plasma concentration can be significant and cause adverse events in patients.

In summary, the present study is the first to investigate the potential DDI between hydromorphone and major cannabinoids and their metabolites using in vitro and IVIVE methods. CBD, 7-OH-CBD, 11-OH-THC, and, to a lesser extent, THC were shown to potently inhibit hydromorphone UGT2B7-mediated metabolism in both recombinant UGT2B7^268His^ and UGT2B7^268Tyr^ microsomes and in HLM. Static mechanistic modeling results suggested that THC, 11-OH-THC, CBD, and 7-OH-CBD would likely cause a DDI to occur in vivo when coadministered with hydromorphone, and subsequent PBPK models investigating the potential DDI between CBD and hydromorphone in both healthy and cirrhotic populations showed a moderate increase in morphine exposure (20%–30%), reaching the FDA and ICHM12 cutoff of an increase in exposure by at least 1.25-fold. Further clinical studies will be needed to fully characterize the DDI in vivo, with dosage adjustments and frequency of administration examined to ensure hydromorphone plasma concentrations remain within its narrow therapeutic window when CBD and hydromorphone are taken together.

## Conflict of interest

The authors declare no conflicts of interest.

## Figures and Tables

**Supplementary Figure 5:**