# Supplementary material for: UGT2B7-mediated drug–drug interaction between cannabinoids and hydromorphone
Source: Drug Metab Dispos. 2025 Jul 24;53(9):100135. doi: 10.1016/j.dmd.2025.100135 (PMC12597549; doi:10.1016/j.dmd.2025.100135)
Supplement: Supplementary Tables 1-3 and Supplementary Figures 1-5 [file mmc1.docx]

**Supplemental Figures**

**Supplemental Figure 1. (A)** Western blot analysis of UGT2B7 variant expression, with lane 1 containing microsomal protein from the parent HEK293 cell line; lane 2, microsomal protein from the rUGT2B7^268His^ overexpressing cell line; lane 3, microsomal protein from the rUGT2B7^268Tyr^ overexpressing cell line. Calnexin was used as a loading control. (**B-D**) Representative Michaelis-Menten curves for hydromorphone metabolism to HM3G in microsomes, with data shown as the mean ± standard deviation of triplicate reactions, from, **(B)** rUGT2B7^268His^ overexpressing cells, **(C)** rUGT2B7^268His^ overexpressing cells, and **(D)** HLM.

**Supplemental Figure 2.** Representative IC_50_ curves for HM3G formation with individual cannabinoids in microsomes from HLM or from microsomes from cell lines overexpressing rUGT2B7^268His^ or rUGT2B7^268Tyr^.

**Supplemental Figure 3.** The observed and simulated plasma concentration–time profiles of hydromorphone in healthy subjects following a single IV dose. The open circles represent the observed data from reported clinical studies (Hill et al., 1991; Drover et al., 2002; Coda et al., 2003; Lohela et al., 2021), with the multiple open circles in the Drover et al. (2002) paper indicative of plasma hydromorphone levels in each participant for each time point. The green and grey lines represent the predicted mean and 5^th^ and 95^th^ percentile concentration – time curve simulated in Simcyp virtual healthy populations.

**Supplemental Figure 4.** The observed and simulated plasma concentration–time profiles of hydromorphone in, **(A)** healthy subjects (Angst et al., 2001; Durnin et al., 2001a; Durnin et al., 2001b; Durnin et al., 2001c; Durnin et al., 2001d; Drover et al., 2002; Toyama et al., 2015; Lohela et al., 2021), and **(B)** subjects with cirrhotic livers (Durnin et al., 2001a) following a single immediate release (IR) oral dose of hydromorphone. The open circles represent the observed data from reported clinical studies, with the multiple open circles in the Drover et al. (2002) paper indicative of plasma hydromorphone levels in each participant for each time point. The green and grey lines represent the predicted mean and 5^th^ and 95^th^ percentile concentration – time curve simulated in Simcyp virtual populations.

**Supplemental Figure 5.** Sensitivity analysis for the change of inhibition constant (K_i_) of CBD on the ratio of simulated hydromorphone AUC. The light blue, orange, and dark blue lines represent the predicted AUC ratio in virtual healthy populations after IV administration of hydromorphone, healthy adults after immediate release administration of hydromorphone, and cirrhotic populations after immediate release administration of hydromorphone, respectively, with increasing potency of CBD K_i_ value. The vertical dotted red line indicates the experimentally determined K_i_ value for CBD against UGT2B7 – mediated hydromorphone glucuronidation. The horizontal black line indicates the FDA AUCR cut off of 1.25.

**Supplemental Table 1.** Estimated maximum plasma concentrations of THC and CBD and their metabolites used to predict the magnitude of *in vivo* cannabinoid drug interactions after consumption of cannabis by either an oral or inhalation route.

| Cannabinoid | Dose (mg)^a^ | | Route of Administration | C_max,hepatic inlet,u_ (µM) |
| --- | --- | --- | --- | --- |
| THC | 20 | Oral | | 0.03 |
|  | 130 | Oral | | 0.20 |
|  | 160 | Oral | | 0.24 |
|  | 25 | Inhalation | | 0.25 |
|  | 70 | Inhalation | | 0.69 |
|  | 100 | Inhalation | | 0.99 |
|  |  |  | |  |
| 11-OH-THC | 20 | Oral | | 0.02 |
|  | 130 | Oral | | 0.10 |
|  | 160 | Oral | | 0.12 |
|  | 25 | Inhalation | | 0.01 |
|  | 70 | Inhalation | | 0.03 |
|  | 100 | Inhalation | | 0.05 |
|  |  |  | |  |
| 11-COOH-THC | 20 | Oral | | 0.19 |
|  | 130 | Oral | | 1.22 |
|  | 160 | Oral | | 1.50 |
|  | 25 | Inhalation | | 0.07 |
|  | 70 | Inhalation | | 0.18 |
|  | 100 | Inhalation | | 0.26 |
|  |  |  | |  |
| CBD | 70 | Oral | | 0.09 |
|  | 700 | Oral | | 0.89 |
|  | 2000 | Oral | | 2.54 |
|  | 19 | Inhalation | | 0.35^c^ |
|  |  |  | |  |
| 7-OH-CBD | 70 | Oral | | 0.03 |
|  | 700 | Oral | | 0.34 |
|  | 2000 | Oral | | 0.98 |
|  | 19 | Inhalation | | N.D.^b^ |

^a^ Doses and C_max_ used to predict AUCR were reported from Bansal et al., 2022. Doses used for modeling 11-OH-THC and 7-OH-CBD were from administered doses of THC and CBD, respectively.

$C_{max,hepatic inlet,u}= f_{u,p}\times\left( C_{max}+ \frac{F_{a}\times F_{g}\times K_{a}\times Dose}{Q_{h}\times R_{b}} \right)$, where f_u,p_ (unbound fraction in plasma) was set to 0.03 (Garrett and Hunt, 1974), F_a_ (fraction absorbed) and F_g_ (dose that escapes gut metabolism) for THC and CBD were set to 1 (FDA Drug Interactions Guidance, 2020), k_a_ for both THC and CBD was set to 0.02 (Cox et al., 2019), Q_H_ (hepatic blood flow) – 1500 mL/min, and BP (blood to plasma ratio) for THC and CBD was set to 0.4 (Schwilke et al., 2009).

^b^ N.D., not determined.

^c^ Doses and C_max_ used to predict AUCR were reported from Cox et al., 2019.

**Supplemental Table 2.** Key physiochemical and system specific input parameters for the development of a physiologically based pharmacokinetic model for cannabidiol using Simcyp v23.

| Parameters | Value |
| --- | --- |
| Physiochemical Properties^a^ |  |
| Molecular weight (g/mol) | 314.5 |
| Log P | 6.33 |
| pK_a_ | 9.13 |
| Blood binding |  |
| Blood to plasma ratio^b^ | 0.67 |
| Fraction unbound in plasma^c^ | 0.013 |
| Absorption^c^ |  |
| First-order absorption model |  |
| f_a_ | 0.25^1^ |
| k_a_ (1/hr) | 0.55^1^ |
| fu_gut_ | 0.013 |
| Q_gut_ (L/hr) | 10.24 |
| Lag time (hr) | 1.5^1^ |
| Distribution: Full PBPK model |  |
| Vss^d^ (L/Kg) | 16.64 |
| Elimination |  |
| Clearance type: Enzyme Kinetic (HLM)^e^ |  |
| CL_int, UGT_ (µL/min/mg of protein) |  |
| UGT1A9 | 541 |
| UGT2B7 | 2162 |
| CL_int, CYP_ (µL/min/mg of protein) |  |
| CYP1A2 | 56 |
| CYP2B6 | 46 |
| CYP2C8 | 51 |
| CYP2C9 | 93 |
| CYP2C19 | 193 |
| CYP2D6 | 38 |
| CYP3A4 | 220 |
| Additional clearance |  |
| CL_R_ (L/hr)^f^ | 0 |
| Auto-inhibition^g^ |  |
| CYP1A2 inactivation |  |
| K_I,u_ (µM) | 0.020 |
| K_inact_ (1/h) | 4.2 |
| CYP2C19 inactivation |  |
| K_I,u_ (µM) | 0.073 |
| K_inact_ (1/h) | 2.4 |
| CYP3A inactivation |  |
| K_I,u_ (µM) | 0.106 |
| K_inact_ (1/h) | 4.7 |

Abbreviation: CL_int_, intrinsic clearance; CL_R_, renal clearance; f_a_, fraction absorbed from dosage form; fu_gut_, fraction unbound in the gut; HLM, human liver microsomes; k_a_, first-order absorption rate constant; K_I,u_, binding-corrected half-maximal inactivation concentration; PBPK, physiologically based pharmacokinetic; pK_a_, dissociation constant; Log P, log of the partition coefficient of a solute between octanol and water; Q_gut_, gut blood flow; Vss, volume of distribution at steady state.

^a^ Physiochemical data was obtained from the ChEMBL database (<https://www.ebi.ac.uk/>).

^b^ Samara et al., 1988.

^c^ Bansal et al., 2022.

^d^ Simcyp predicted.

^e^ Bansal et al., 2023.

^f^ Tayo et al., 2020.

^g^ Bansal et al., 2020.

^1^ Fasted.

**Supplemental Table 3.** PBPK model: predicted and observed (mean and 90% CI) hydromorphone exposure in studies of healthy and cirrhotic adults after a single intravenous or immediate release oral dose of hydromorphone.

| Study | Population | Age range | % female | | Population size | | Dose^a^ (mg)  and route of administration | | Parameters | | Obs | | Pred | | Pred/Obs | |
| --- | --- | --- | --- | --- | --- | --- | --- | --- | --- | --- | --- | --- | --- | --- | --- | --- |
| Lohela et al., 2021 | Healthy | 20 – 34 | 0.42 | | 12 subjects x  34 trials | | 0.02 mg/kg IV | | AUC (ng•h•mL^-1^) | | 11.85 | | 17.39 | | 1.47 | |
| Drover et al., 2002 | Healthy | 21 – 34 | 0.5 | | 12 subjects x  34 trials | | 8 IV | | AUC (ng•h•mL^-1^) | | 92.81 | | 92.61 | | 1.00 | |
| Hill et al., 1991 | Healthy | 21 – 38 | 0 | | 10 subjects x 40 trials | | 0.01 mg/kg IV | | AUC (ng•h•mL^-1^) | | 8.33 | | 7.69 | | 0.92 | |
| Hill et al., 1991 | Healthy | 21 – 38 | 0 | | 10 subjects x 40 trials | | 0.02 mg/kg IV | | AUC (ng•h•mL^-1^) | | 12.62 | | 15.38 | | 1.22 | |
| Hill et al., 1991 | Healthy | 21 – 38 | 0 | | 10 subjects x 40 trials | | 0.04 mg/kg IV | | AUC (ng•h•mL^-1^) | | 21.98 | | 30.77 | | 1.40 | |
| Coda et al., 2003 | Healthy | 20- 31 | 0.46 | | 24 subjects x  17 trials | | 2 IV | | AUC (ng•h•mL^-1^) | | 17.22 | | 21.78 | | 1.26 | |
| **Average GMFEs (range)** | |  |  | |  | |  | | AUC | | 1.99 (1.00– 2.56) | | | | | |
| **Average MRDs (range)** | |  |  | |  | |  | | AUC | | 1.82 (1.00 – 1.47) | | | | | |
| Lohela et al., 2021 | Healthy | 20 – 34 | 0.42 | | 12 subjects x  34 trials | | 2.6 IR | | AUC (ng•h•mL^-1^) | | 6.60 | | 4.98 | | 0.75 | |
|  |  |  |  | |  | |  | | C_max_ (ng•mL^-1^) | | 1.48 | | 1.78 | | 1.20 | |
| Drover et al., 2002 | Healthy | 21 – 34 | 0.5 | | 12 subjects x  34 trials | | 8 IR | | AUC (ng•h•mL^-1^) | | 15.83 | | 15.54 | | 0.98 | |
|  |  |  |  | |  | |  | | C_max_ (ng•mL^-1^) | | 5.19 | | 5.89 | | 1.13 | |
| Angst et al., 2001 | Healthy | 21 – 34 | 0.5 | | 12 subjects x  34 trials | | 8 IR | | AUC (ng•h•mL^-1^) | | 7.35 | | 13.32 | | 1.81 | |
|  |  |  |  | |  | |  | | C_max_ (ng•mL^-1^) | | 4.74 | | 5.89 | | 1.24 | |
| Toyama et al., 2015 | Healthy | 32 – 43 | 0 | | 6 subjects x 67 trials | | 1 IR | | AUC (ng•h•mL^-1^) | | 1.8 | | 2.07 | | 1.15 | |
|  |  |  |  | |  | |  | | C_max_ (ng•mL^-1^) | | 0.7 | | 0.75 | | 1.07 | |
| Toyama et al., 2015 | Healthy | 32 – 43 | 0 | | 6 subjects x 67 trials | | 2 IR | | AUC (ng•h•mL^-1^) | | 4.1 | | 4.15 | | 1.01 | |
|  |  |  |  | |  | |  | | C_max_ (ng•mL^-1^) | | 1.0 | | 1.50 | | 1.5 | |
| Toyama et al., 2015 | Healthy | 32 – 43 | 0 | | 6 subjects x 67 trials | | 4 IR | | AUC (ng•h•mL^-1^) | | 10.3 | | 8.30 | | 0.81 | |
|  |  |  |  | |  | |  | | C_max_ (ng•mL^-1^) | | 2.0 | | 3.0 | | 1.5 | |
| Durnin et al., 2001a | Healthy | 43 – 59 | 0.25 | | 12 subjects x  34 trials | | 4 IR | | AUC (ng•h•mL^-1^) | | 10.4 | | 8.54 | | 0.82 | |
|  |  |  |  | |  | |  | | C_max_ (ng•mL^-1^) | | 2.0 | | 3.06 | | 1.53 | |
| Durnin et al., 2001b | Healthy | 19 – 30 | 1 | | 18 subjects x  23 trials | | 8 IR | | AUC (ng•h•mL^-1^) | | 13.4 | | 16.60 | | 1.24 | |
|  |  |  |  | |  | |  | | C_max_ (ng•mL^-1^) | | 5.12 | | 6.65 | | 1.30 | |
| Durnin et al., 2001b | Healthy | 24 – 39 | 0 | | 18 subjects x  23 trials | | 8 IR | | AUC (ng•h•mL^-1^) | | 13.0 | | 14.52 | | 1.12 | |
|  |  |  |  | |  | |  | | C_max_ (ng•mL^-1^) | | 4.09 | | 5.24 | | 1.28 | |
| Durnin et al., 2001c | Healthy | 19 – 33 | 0.5 | | 18 subjects x  23 trials | | 4 IR | | AUC (ng•h•mL^-1^) | | 6.1 | | 7.77 | | 1.27 | |
|  |  |  |  | |  | |  | | C_max_ (ng•mL^-1^) | | 2.49 | | 2.95 | | 1.18 | |
| Durnin et al., 2001d | Healthy | 21 – 42 | 0 | | 26 subjects x  16 trials | | 2 IR | | AUC (ng•h•mL^-1^) | | 4.28 | | 3.65 | | 0.85 | |
|  |  |  |  | |  | |  | | C_max_ (ng•mL^-1^) | | 1.25 | | 1.32 | | 1.06 | |
| Durnin et al., 2001d | Healthy | 21 – 42 | 0 | | 26 subjects x  16 trials | | 4 IR | | AUC (ng•h•mL^-1^) | | 7.94 | | 7.30 | | 0.92 | |
|  |  |  |  | |  | |  | | C_max_ (ng•mL^-1^) | | 2.50 | | 2.63 | | 1.05 | |
| Durnin et al., 2001d | Healthy | 21 – 42 | 0 | | 25 subjects x  16 trials | | 8 IR | | AUC (ng•h•mL^-1^) | | 15.0 | | 14.60 | | 0.97 | |
|  |  |  |  | |  | |  | | C_max_ (ng•mL^-1^) | | 5.38 | | 5.27 | | 0.98 | |
| **Average GMFEs (range)** | |  |  |  | |  | | AUC | | 1.89 (1.16 – 3.22) | | | | | |  |
|  |  |  |  |  | |  | | C_max_ | | 2.29 (1.02 – 1.81) | | | | | |  |
| **Average MRDs (range)** | |  |  |  | |  | | AUC | | 1.98 (1.24 – 2.69) | | | | | |  |
|  |  |  |  |  |  |  | | C_max_ | | 2.41 (1.02 – 1.53) | | | | | |  |
| Durnin et al., 2001a | Moderate  Cirrhosis | 45 – 60 | 0.25 | 12 subjects x  34 trials | | 4 IR | | AUC (ng•h•mL^-1^) | | 41.8 | | 35.15 | | 0.84 | |  |
|  |  |  |  |  |  |  |  | C_max_ (ng•mL^-1^) | | 8.3 | | 8.37 | | 1.01 | |  |
| **GMFEs** | |  |  |  | |  | | AUC | | 1.88 | | | | | |  |
|  |  |  |  |  | |  | | C_max_ | | 1.16 | | | | | |  |
| **MRDs** | |  |  |  | |  | | AUC | | 1.19 | | | | | |  |
|  |  |  |  |  |  |  | | C_max_ | | 1.01 | | | | | |  |

Abbreviations: GMFE – Geometric mean fold error; MRD – Mean relative deviation; Obs – observed; Pred – Predicted; # females – proportion of females in the study; Pred/Obs – predicted/observed ratio; IV – intravenous; IR – immediate release; AUC – area under the plasma concentration – time curve; C_max_ – maximum plasma concentration.

^a^ Actual free-based dose

**References (in the order that they were cited above)**

Hill, H.F., et al., *Multiple-dose evaluation of intravenous hydromorphone pharmacokinetics in normal human subjects.* Anesth Analg, 1991. **72**(3): p. 330-6.

Drover, D.R., et al., *Input characteristics and bioavailability after administration of immediate and a new extended-release formulation of hydromorphone in healthy volunteers.* Anesthesiology, 2002. **97**(4): p. 827-36.

Coda, B.A., et al., *Pharmacokinetics and bioavailability of single-dose intranasal hydromorphone hydrochloride in healthy volunteers.* Anesth Analg, 2003. **97**(1): p. 117-23, table of contents.

Lohela, T.J., et al., *Rifampin Reduces the Plasma Concentrations of Oral and Intravenous Hydromorphone in Healthy Volunteers.* Anesth Analg, 2021. **133**(2): p. 423-434.

Angst, M.S., et al., *Pharmacodynamics of orally administered sustained- release hydromorphone in humans.* Anesthesiology, 2001. **94**(1): p. 63-73.

Durnin, C., et al., *Pharmacokinetics of oral immediate-release hydromorphone (Dilaudid IR) in subjects with moderate hepatic impairment.* Proc West Pharmacol Soc, 2001. **44**: p. 83-4.

Durnin, C., et al., *Pharmacokinetics of oral immediate-release hydromorphone (Dilaudid IR) in male and female subjects.* Proc West Pharmacol Soc, 2001. **44**: p. 77-8.

Durnin, C., et al., *Pharmacokinetics of oral immediate-release hydromorphone (Dilaudid IR) in young and elderly subjects.* Proc West Pharmacol Soc, 2001. **44**: p. 79-80.

Durnin, C., et al., *Dose proportionality of the pharmacokinetics of oral immediate-release hydromorphone (Dilaudid IR).* Proc West Pharmacol Soc, 2001. **44**: p. 73-4.

Toyama, K., et al., *Single-dose evaluation of safety, tolerability and pharmacokinetics of newly formulated hydromorphone immediate-release and hydrophilic matrix extended-release tablets in healthy Japanese subjects without co-administration of an opioid antagonist.* J Clin Pharmacol, 2015. **55**(9): p. 975-84.

Bansal, S., M.F. Paine, and J.D. Unadkat, *Comprehensive Predictions of Cytochrome P450 (P450)-Mediated In Vivo Cannabinoid-Drug Interactions Based on Reversible and Time-Dependent P450 Inhibition in Human Liver Microsomes.* Drug Metab Dispos, 2022. **50**(4): p. 351-360.

Garrett, E.R. and C.A. Hunt, *Physiochemical properties, solubility, and protein binding of delta9-tetrahydrocannabinol.* J Pharm Sci, 1974. **63**(7): p. 1056-64.

FDA, F.a.D.A., *In Vitro Drug Interaction Studies - Cytochrome P450 Enzyme - and Transporter-Mediated Drug Interactions Guidance for Industry*, U.S.D.o.H.a.H. Services, Editor. 2020, Center for Drug Evaluation and Research, Food and Drug Administration. p. 1-46.

Cox, E.J., et al., *A marijuana-drug interaction primer: Precipitants, pharmacology, and pharmacokinetics.* Pharmacol Ther, 2019. **201**: p. 25-38.

Schwilke, E.W., et al., *Δ9-Tetrahydrocannabinol (THC), 11-Hydroxy-THC, and 11-Nor-9-carboxy-THC Plasma Pharmacokinetics during and after Continuous High-Dose Oral THC.* Clinical Chemistry, 2009. **55**(12): p. 2180-2189.

Samara, E., M. Bialer, and R. Mechoulam, *Pharmacokinetics of cannabidiol in dogs.* Drug Metab Dispos, 1988. **16**(3): p. 469-72.

Bansal, S., et al., *A Physiologically-Based Pharmacokinetic Model for Cannabidiol in Healthy Adults, Hepatically-Impaired Adults, and Children.* Drug Metabolism and Disposition, 2023. **51**(6): p. 743-752.

Tayo, B., et al., *A Phase I, Open-Label, Parallel-Group, Single-Dose Trial of the Pharmacokinetics, Safety, and Tolerability of Cannabidiol in Subjects with Mild to Severe Renal Impairment.* Clin Pharmacokinet, 2020. **59**(6): p. 747-755.

Bansal, S., et al., *Predicting the Potential for Cannabinoids to Precipitate Pharmacokinetic Drug Interactions via Reversible Inhibition or Inactivation of Major Cytochromes P450.* Drug Metab Dispos, 2020. **48**(10): p. 1008-1017.
